# Subgroup Identification in Alzheimer's Disease Trial

**DOI:** 10.1002/alz70856_099562

**Published:** 2025-12-24

**Authors:** Lei Liu

**Affiliations:** ^1^ Division of Biostatistics, Washington University in St Louis, St Louis, MO, USA

## Abstract

**Background:**

Alzheimer's disease (AD) and its precursor, mild cognitive impairment (MCI), present significant challenges due to heterogeneous progression and treatment responses. Identifying subgroups of patients who benefit from specific treatments, such as Donepezil, is critical for advancing precision medicine in AD management.

**Method:**

The ITree‐MMRM framework was developed by integrating the Interaction Tree (ITree) method with the Mixed Model for Repeated Measures (MMRM) to identify patient subgroups with heterogeneous long‐term treatment effects. The approach was applied to a three‐year clinical trial on Donepezil treatment in 769 MCI patients, using changes in Clinical Dementia Rating Scale Sum of Boxes (CDR‐SOB) scores as the primary outcome. Bootstrap pruning was utilized to mitigate overoptimism in subgroup identification.

**Result:**

The ITree‐MMRM framework identified an MCI subgroup (MMSE score ≤24) that exhibited significant long‐term benefits from Donepezil at 36 months, with improved CDR‐SOB scores. Subgroups with higher baseline cognitive scores showed less benefit. Merging subgroups increased the treatment's applicability but slightly reduced the observed effect size. Simulation studies demonstrated the framework's robustness and superiority over existing methods in subgroup detection and handling complex correlation structures.

**Conclusion:**

The ITree‐MMRM framework successfully identified MCI subgroups with differential long‐term responses to Donepezil, providing insights into targeted treatment strategies for AD progression. These findings emphasize the potential of personalized medicine in improving therapeutic outcomes for MCI and AD, warranting further validation in larger clinical trials.

